# Supplementary material for: NTBC Treatment Monitoring in Chilean Patients with Tyrosinemia Type 1 and Its Association with Biochemical Parameters and Liver Biomarkers
Source: J Clin Med. 2021 Dec 13;10(24):5832. doi: 10.3390/jcm10245832 (PMC8706240; doi:10.3390/jcm10245832)
Supplement: Supplementary file 1 [file jcm-10-05832-s001.zip › jcm-1396947-supplementary.pdf]

Supplementary Materials:

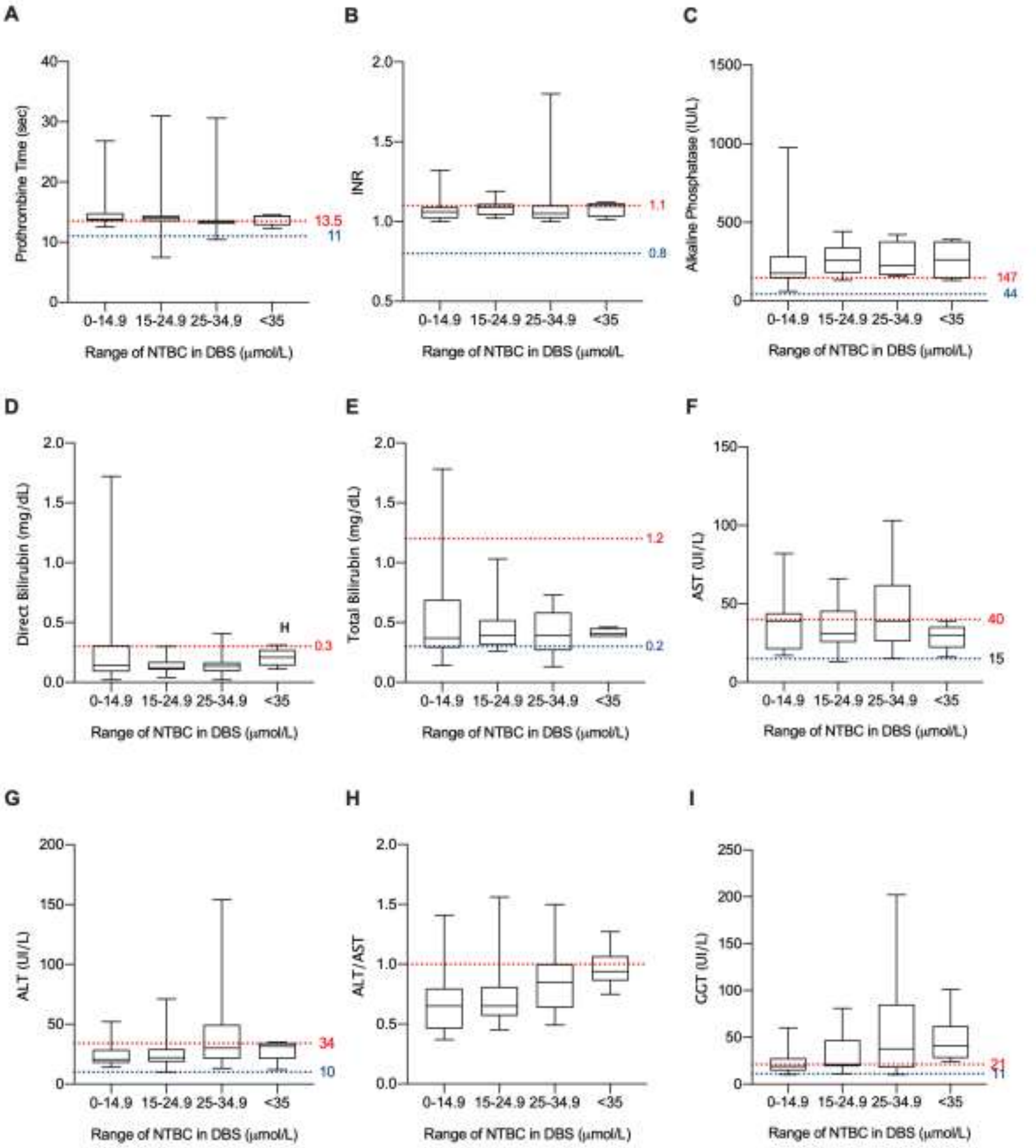

Figure S1. Liver biomarkers in association with NTBC plasma concentration. Amino acids variation according to NTBC concentration ranges. Boxplots of Prothrombin time (**A**); INR (**B**); Alkaline Phosphatase (**C**); Direct bilirubin (**D**); Total bilirubin (**E**); AST (**F**); ALT (**G**); ALT/AST (**H**); GGT (**I**) by NTBC concentration ranges. Each boxplot represents the 25, 50, 75 quartiles for each amino acid. Dotted lines represent maximum and minimum allowable concentrations according to our reference laboratory: prothrombin time: 11-13.5 sec; INR: 0.8-1.1; alkaline phosphatase: 46-147 UI/L; direct bilirubin: <0.3 mg/dL; total bilirubin: 0.2-1.2 mg/dL; AST: 15-40 UI/L; ALT: 10-34 UI/L; GGT: 11-21 UI/L. INR: international normalized ratio; AST: aspartate aminotransferase; ALT: alanine transaminase; GGT: gamma-glutamyl transferase.
